# Quantification of Amino Acid Enantiomers Using Electrospray Ionization and Ultraviolet Photodissociation

**DOI:** 10.5702/massspectrometry.A0097

**Published:** 2021-09-07

**Authors:** Kanako Inoue, Akimasa Fujihara

**Affiliations:** 1 Department of Chemistry, Graduate School of Science, Osaka Prefecture University, Osaka 599–8531, Japan

**Keywords:** chiral recognition, hydrogen bond, quantitative analysis, ion trap

## Abstract

The enantioselectivity of tryptophan (Trp) for amino acids, such as alanine (Ala), valine (Val), and serine (Ser), was investigated using ultraviolet (UV) photoexcitation and tandem mass spectrometry. Product ion spectra of cold gas-phase amino acid enantiomers that were hydrogen-bonded to Na^+^(L-Trp) were measured using a variable-wavelength UV laser and a tandem mass spectrometer equipped with an electrospray ionization source and a cold ion trap. Na^+^(L-Trp), formed *via* amino acid detachment, and the elimination of CO_2_ from the clusters were observed in the product ion spectra. For photoexcitation at 265 nm, the relative abundance of Na^+^(L-Trp) compared to that of the precursor ion observed in the product ion spectrum of heterochiral Na^+^(L-Trp)(D-Ala) was larger than that observed in the product ion spectrum of homochiral Na^+^(L-Trp)(L-Ala). A difference between the Val enantiomers in the relative abundance of the precursor and product ions was observed in the case of photoexcitation at 272 nm. The elimination of CO_2_ was not observed for L-Ser for the 285 nm photoexcitation, which was the main reaction pathway for D-Ser. Photoexcited Trp chiral recognition was applied to identify and quantify the amino acid enantiomers in solution. Ala, Val, and Ser enantiomers in solution were quantified from their relative abundances in single product ion spectra measured using photoexcitation at 265, 272, and 285 nm, respectively, for hydrogen-bonded Trp within the clusters.

## INTRODUCTION

The identification of amino acid enantiomers is critical in the field of biochemistry, because D-amino acids play significant roles in living organisms.^1,2)^ Amino acid enantiomers are distinguished using analytical methods based on chromatography, nuclear magnetic resonance (NMR) spectroscopy, and X-ray crystallography.^3)^ Mass spectrometry is widely used for analyses of molecular structures because it is highly sensitive, selective, and suitable for analyzing mixtures and for detecting amino acid mutations in peptides.^4,5)^ The identification of amino acid enantiomers is also critical in the field of mass spectrometry, and several mass spectrometry-based techniques for this have been developed over the past two decades.^6,7)^ Collision-activated dissociation and ion mobility-mass spectrometry of gas-phase, copper-bound complexes is used to distinguish amino acid enantiomers by detecting variations in enantiomer binding energies and collision cross-sections, respectively.^8,9)^ NMR spectroscopy of gas-phase ions using a magnetic resonance acceleration technique has also been proposed.^10)^

The chiral recognition of biomolecules is attributed to the homochirality in biomolecules consisting of L-amino acids and D-sugars. The origin of biomolecular homochirality in living organisms is clearly one of the most scientifically important issues of our time. The extraterrestrial origin of enantiomeric excess in amino acids has been evaluated in numerous studies on abiotic amino acid syntheses in simulated interstellar ice,^11–13)^ amino acid analyses of meteorites,^14,15)^ and enantioselective destruction by circularly polarized light.^16,17)^

The remarkable homochiral preference of gas-phase serine (Ser) octamers has been investigated using mass spectrometry-based techniques.^18–21)^ Gas-phase proline clusters also exhibited a homochiral preference.^22,23)^ The physical and chemical properties of cold gas-phase hydrogen-bonded clusters have been investigated as a model for chemical evolution in interstellar molecular clouds because chemical reactions in space occur at low temperatures and densities.^24)^ D-Tryptophan (D-Trp), when photoexcited at 266 nm, undergoes dissociation *via* CO_2_ loss when it was noncovalently complexed with L-Ser or L-threonine (L-Thr) in the presence of Na^+^, whereas such an enantioselective reaction was not observed for the noncovalent complex with alanine (Ala).^25)^ This indicates that the side-chain OH group of the molecule contributed to the enantioselective photodissociation.

For hydrophilic amino acids such as Ser, Thr, glutamine, asparagine, glutamic acid, and aspartic acid, enantioselective dissociation induced by 266 nm photoexcitation was observed in the product ion spectra of hydrogen-bonded clusters with Na^+^(L-Trp).^25,26)^ In the case of a hydrophobic amino acid such as Ala, enantioselective dissociation was not observed and its enantiomers could not be discriminated.^25)^ This is one of the limitations in the chiral discrimination of amino acids when photoexcitation at 266 nm is used, which was the fourth harmonic of a compact and inexpensive Nd:YAG laser.

In this study, amino acid enantiomer selectivity was investigated using the ultraviolet (UV) photoexcitation of Trp in cold gas-phase hydrogen-bonded clusters containing hydrophobic amino acids, such as Ala and valine (Val), in addition to Ser, a hydrophilic amino acid. Hydrogen-bonded Trp chiral recognition in cold gas-phase clusters generated *via* electrospray ionization and collisional cooling was used in the identification and quantification of amino acid enantiomers in solution.

## EXPERIMENTAL

Product ion spectra of hydrogen-bonded clusters of amino acid enantiomers were measured using a wavelength-variable UV laser and a home-built tandem mass spectrometer equipped with an electrospray ionization source and a cold ion trap.^27)^ Hydrogen-bonded clusters of amino acid enantiomers with Na^+^(L-Trp) were generated *via* the electrospray ionization of 1.0 mM solutions that contained L-Trp, NaCl, and analyte molecules in a mixture of water and methanol. The ionization source was operated at a sample flow rate of 3 μL/min, a sheath gas flow rate of 3 L/min, and an applied voltage of 2 kV. The hydrogen-bonded cluster ions were transferred to the gas phase through a metal capillary and octopole ion guide. The gas-phase ions were mass-selected using a quadrupole mass filter and thermalized in a cold ion trap (8 K) *via* multiple collisions with He buffer gas. The mass-selected, temperature-controlled ions were then irradiated with a photoexcitation laser pulse at 1.2 mJ/pulse, which was unfocused so as to allow spatial overlap between the ion packets and laser pulse. The product ion masses were determined using a reflectron time-of-flight spectrometer. The ion signals were quantified using dual microchannel plates (F4655, Hamamatsu Photonics, Hamamatsu, Japan) and a digital storage oscilloscope (104MXi, Teledyne LeCroy, Chestnut Ridge, NY, USA). The photoexcitation laser pulse was the third harmonic of a tunable Ti:sapphire laser (LT-2211T, LOTIS TII, Minsk, Belarus) pumped by the second harmonic of a Nd:YAG laser (LS-2145TF, LOTIS TII).

## RESULTS AND DISCUSSION

### UV photoexcitation of hydrogen-bonded clusters

**Figure 1:**
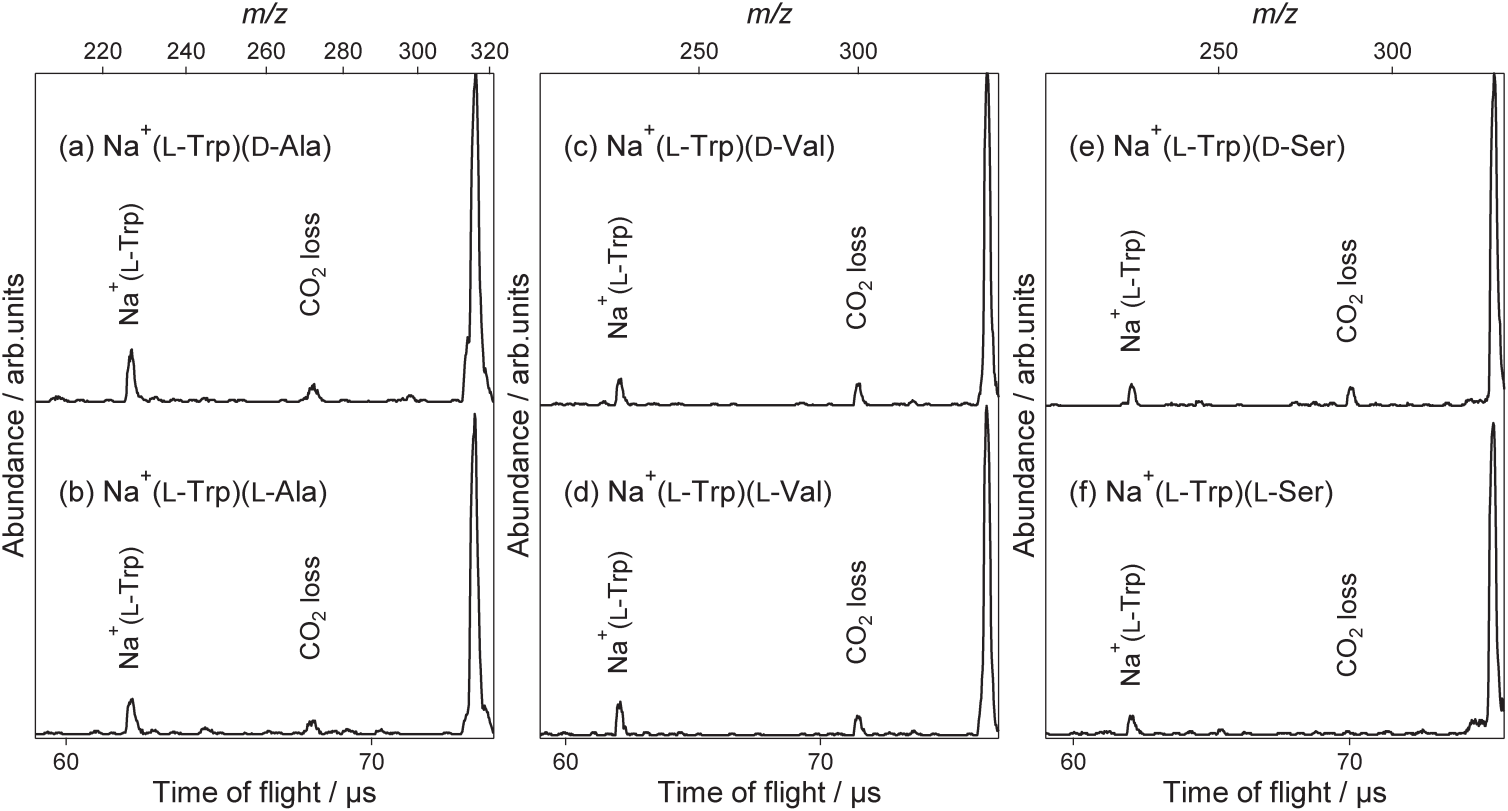
Fig. 1. Product ion spectra measured by photoexcitation at 265 nm at 8 K for (a) Na^+^(L-Trp)(D-Ala), (b) Na^+^(L-Trp)(L-Ala), (c) Na^+^(L-Trp)(D-Val), (d) Na^+^(L-Trp)(L-Val), (e) Na^+^(L-Trp)(D-Ser), and (f) Na^+^(L-Trp)(L-Ser). The spectra were normalized using precursor ion abundances.

Product ion spectra measured using 265 nm photoexcitation at 8 K of Ala, Val, or Ser enantiomers hydrogen-bonded with Na^+^(L-Trp) are shown in Fig. 1. Na^+^(L-Trp), formed *via* amino acid detachment, and the loss of CO_2_ from the clusters were observed. The loss of CO_2_ from non-zwitterionic amino acids with NH_2_ and COOH groups was reported during UV photoexcitation studies of an ice matrix.^28)^ The zwitterionic Trp with NH_3_^+^ and COO^−^ groups dissociated *via* the loss of NH_3_.^29)^ Therefore, the product ion spectra shown in Fig. 1 indicate that the amino acids within the clusters are non-zwitterionic structures that contain NH_2_ and COOH groups.

The relative abundance of Na^+^(L-Trp) (*m*/*z* 227) in the product ion spectrum of heterochiral Na^+^(L-Trp)(D-Ala) (*m*/*z* 316) is larger than that in the product ion spectrum of homochiral Na^+^(L-Trp)(L-Ala). The product ion spectra of heterochiral Na^+^(D-Trp)(L-Ala) and homochiral Na^+^(D-Trp)(D-Ala) were identical with the spectra of heterochiral Na^+^(L-Trp)(D-Ala) and homochiral Na^+^(L-Trp)(L-Ala), respectively. Ala enantiomers are identified by the relative photodissociation cross sections of the cold gas-phase hydrogen-bonded clusters. In contrast, no differences between Val enantiomers were observed in the product ion spectra, as shown in Figs. 1c and d. In the Ser product ion spectra, the relative abundance of the CO_2_-eliminated ion (*m*/*z* 288) compared to that of Na^+^(L-Trp) for heterochiral Na^+^(L-Trp)(D-Ser) (*m*/*z* 332) is larger than that for homochiral Na^+^(L-Trp)(L-Ser). Ser enantiomers are identified by the photoinduced elimination of CO_2_, as previously reported.^25)^ Therefore, different enantioselective phenomena are observed within cold gas-phase hydrogen-bonded clusters containing Ala, Val, or Ser.

### Photoexcitation wavelength and enantioselectivity

**Figure 2:**
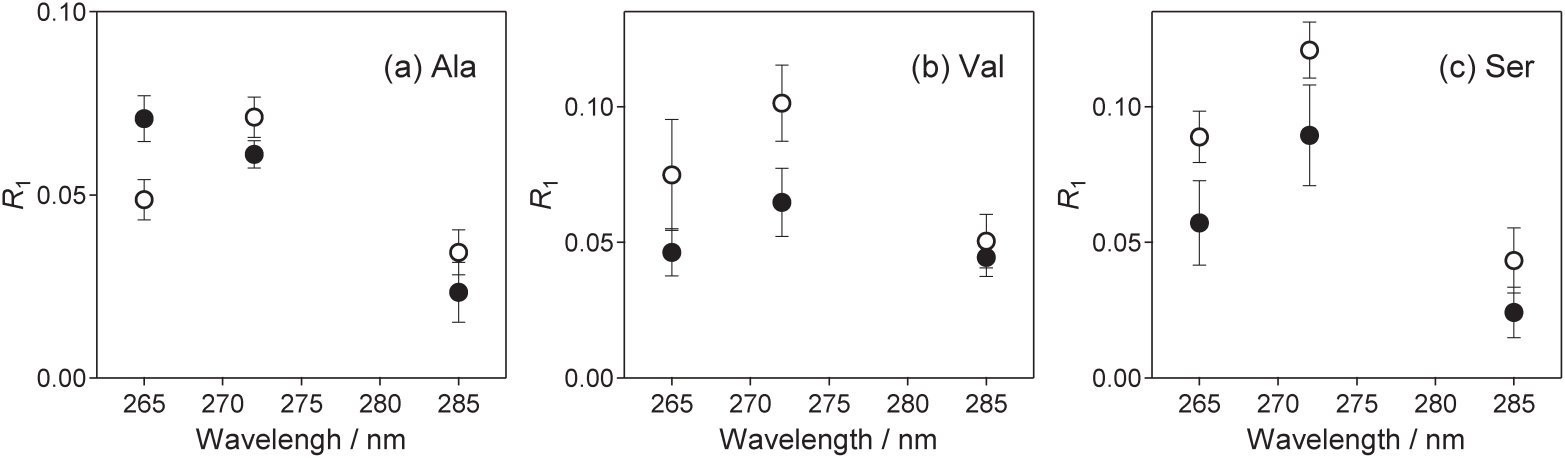
Fig. 2. Photoexcitation-wavelength dependence of the relative abundance ratio *R*_1_ of (a) Ala, (b) Val, and (c) Ser enantiomers. *R*_1_ is derived by dividing the abundance of Na^+^(L-Trp) by the abundance of the precursor ion in the product ion spectrum of the hydrogen-bonded cluster with Na^+^(L-Trp) at 8 K. D- and L-Amino acids are indicated by closed and open circles, respectively. Error bars represent standard deviations.

To investigate the effect of photoexcitation wavelength on the enantioselectivities, UV photoexcitation studies were conducted on the cold gas-phase hydrogen-bonded clusters in the wavelength range of the ππ* state of the Trp indole ring. The photoexcitation wavelengths used were 265, 272, and 285 nm. It has been reported that photoexcitation at 272 and 285 nm can be used to distinguish isomeric amino acids and pentoses, respectively.^30,31)^ In the case where the cold gas-phase hydrogen-bonded clusters were photoexcited at 272 and 285 nm, Na^+^(L-Trp), formed *via* amino acid detachment, and the loss of CO_2_ from the clusters were observed in the product ion spectra as in the case of photoexcitation at 265 nm (Fig. 1). The dependence of photoexcitation wavelength on the photodissociation of the Ala, Val, and Ser enantiomers are displayed in Fig. 2 with the D- and L-amino acids plotted using closed and open circles, respectively. The relative abundance ratio *R*_1_ is derived by dividing the abundance of the Na^+^(L-Trp) by the abundance of the precursor ion in the product ion spectrum of the hydrogen-bonded cluster with Na^+^(L-Trp). As shown in Fig. 2a, the difference in *R*_1_ values between the Ala enantiomers observed in the product ion spectra of photoexcitation at 265 nm (Figs. 1a and b) decreases at 272 and 285 nm. In contrast, the difference in *R*_1_ values between Val enantiomers is largest for photoexcitation at 272 nm. Hence, photoexcitations at 265 nm and 272 nm are optimal for distinguishing Ala and Val enantiomers, respectively.

**Figure 3:**
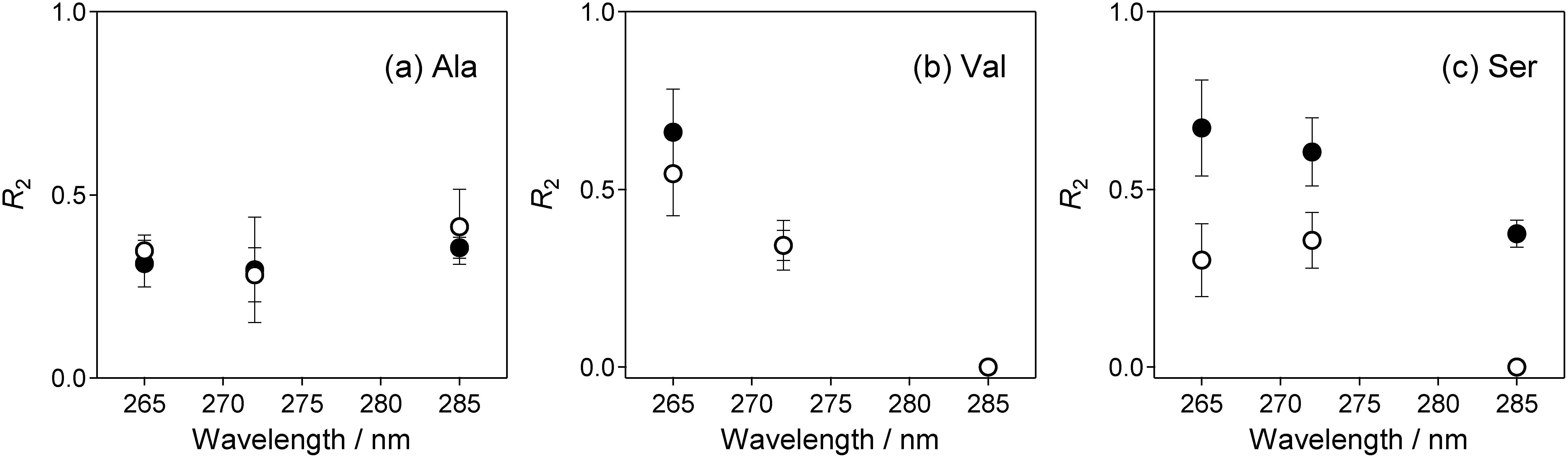
Fig. 3. Photoexcitation-wavelength dependence of the relative abundance ratio *R*_2_ of (a) Ala, (b) Val, and (c) Ser enantiomers. *R*_2_ is derived by dividing the abundance of the CO_2_-eliminated ion by the abundance of Na^+^(L-Trp) in the product ion spectrum of the hydrogen-bonded cluster with Na^+^(L-Trp) at 8 K. D- and L-Amino acids are indicated by closed and open circles, respectively. Error bars represent standard deviations.

Ser enantiomers display the same tendency in the photoexcitation-wavelength dependence of *R*_1_, as displayed in Fig. 2c. To examine the effects of the photoexcitation wavelength on the enantioselective reaction, the relative abundance ratios *R*_2_ for Ala, Val, and Ser enantiomers were derived by dividing the abundance of the CO_2_-eliminated ion by the abundance of Na^+^(L-Trp) in the product ion spectrum. The *R*_2_ values are plotted as a function of photoexcitation wavelength in Fig. 3. For the clusters of Ala enantiomers that are hydrogen-bonded with Na^+^(L-Trp), Na^+^(L-Trp), formed *via* the detachment of Ala, and the elimination of CO_2_ from the clusters can be observed in the product ion spectra in the case of photoexcitation at 265, 272, and 285 nm. In addition, *R*_2_ does not depend on the photoexcitation wavelength. For Val enantiomers, CO_2_ elimination from the clusters is not observed in the product ion spectra for the 285 nm photoexcitation, which is the main reaction pathway in the case of photoexcitation at 265 and 272 nm. However, no differences in *R*_2_ values were observed between Val enantiomers at these wavelengths. The enantiomers in the hydrogen-bonded clusters containing Ala or Val exhibited the same photoreactivity and wavelength dependence, thus making them indistinguishable using *R*_2_. The relative abundance of the 265 nm photoinduced CO_2_ elimination for D-Ser was larger than that of the 265 nm photoinduced CO_2_ elimination for L-Ser, as shown in Figs. 1e, 1f, and 3c. In contrast, no CO_2_ elimination was observed for L-Ser at photoexcitation of 285 nm, which is the main pathway for D-Ser. Thus, the enantioselectivities and photoexcitation-wavelength dependences are different for Ala, Val, and Ser.

Wavelength dependence of enantioselective reactions was reported for cold gas-phase hydrogen-bonded clusters of Trp and carbohydrate enantiomers. The enantioselective reactions observed at 265–280 nm were caused by a photoinduced electron transfer from the indole ring to the carboxyl group of Trp.^32)^ The ππ* state of the Trp indole ring observed at 285 nm contributed to the recognition of chiral pentose units.^31)^ For the chiral recognition between amino acids reported here, the hydrogen-bonding and electronic structures of Trp within the amino acid clusters may be critical, as in the case of the carbohydrates. A knowledge of the geometric and electronic structures of the cold gas-phase hydrogen-bonded clusters is therefore crucial for developing an understanding of the molecular recognition mechanism responsible, which could be further studied using photodissociation spectroscopy, ion mobility spectrometry, and theoretical calculations.

### Quantification of amino acid enantiomers in solution

Ala and Val enantiomers were identified by the relative photodissociation cross sections in the case of photoexcitation at 265 and 272 nm, respectively, whereas Ser enantiomers could be identified by photoinduced CO_2_ elimination. Hydrogen-bonded Trp chiral recognition was applied to the quantification of amino acid enantiomers in solution *via* the UV photoexcitation of cold gas-phase hydrogen-bonded clusters with Na^+^(L-Trp) generated *via* electrospray ionization and collisional cooling.

**Figure 4:**
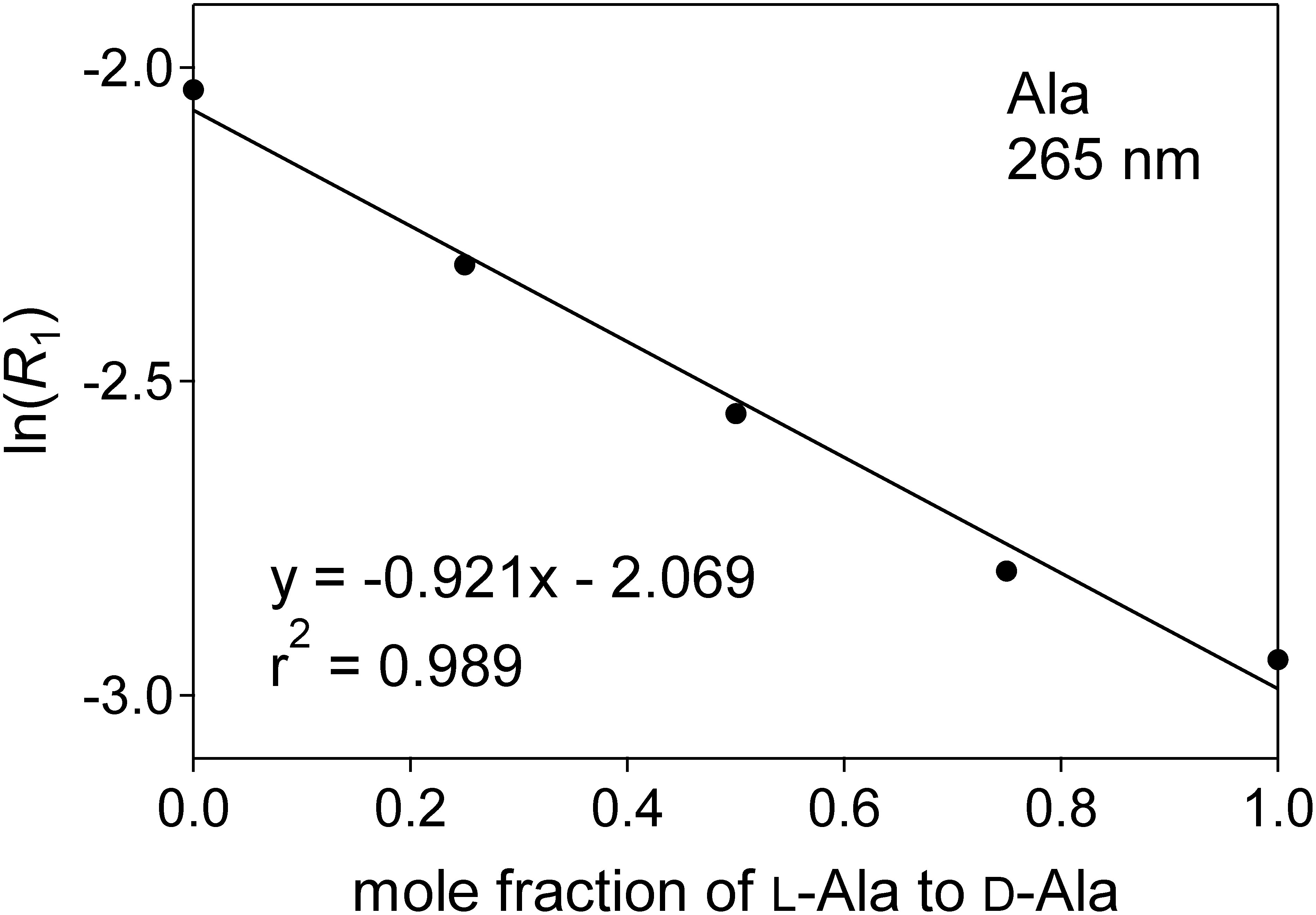
Fig. 4. Linear plot of ln(*R*_1_) for Na^+^(L-Trp)(Ala) at 8 K *vs.* the mole fraction of L-Ala to D-Ala in solution. The relative abundance ratio *R*_1_ is derived by dividing the abundance of Na^+^(L-Trp) by the abundance of the precursor ion in the product ion spectrum measured by photoexcitation at 265 nm at 8 K for Na^+^(L-Trp)(Ala).

**Figure 5:**
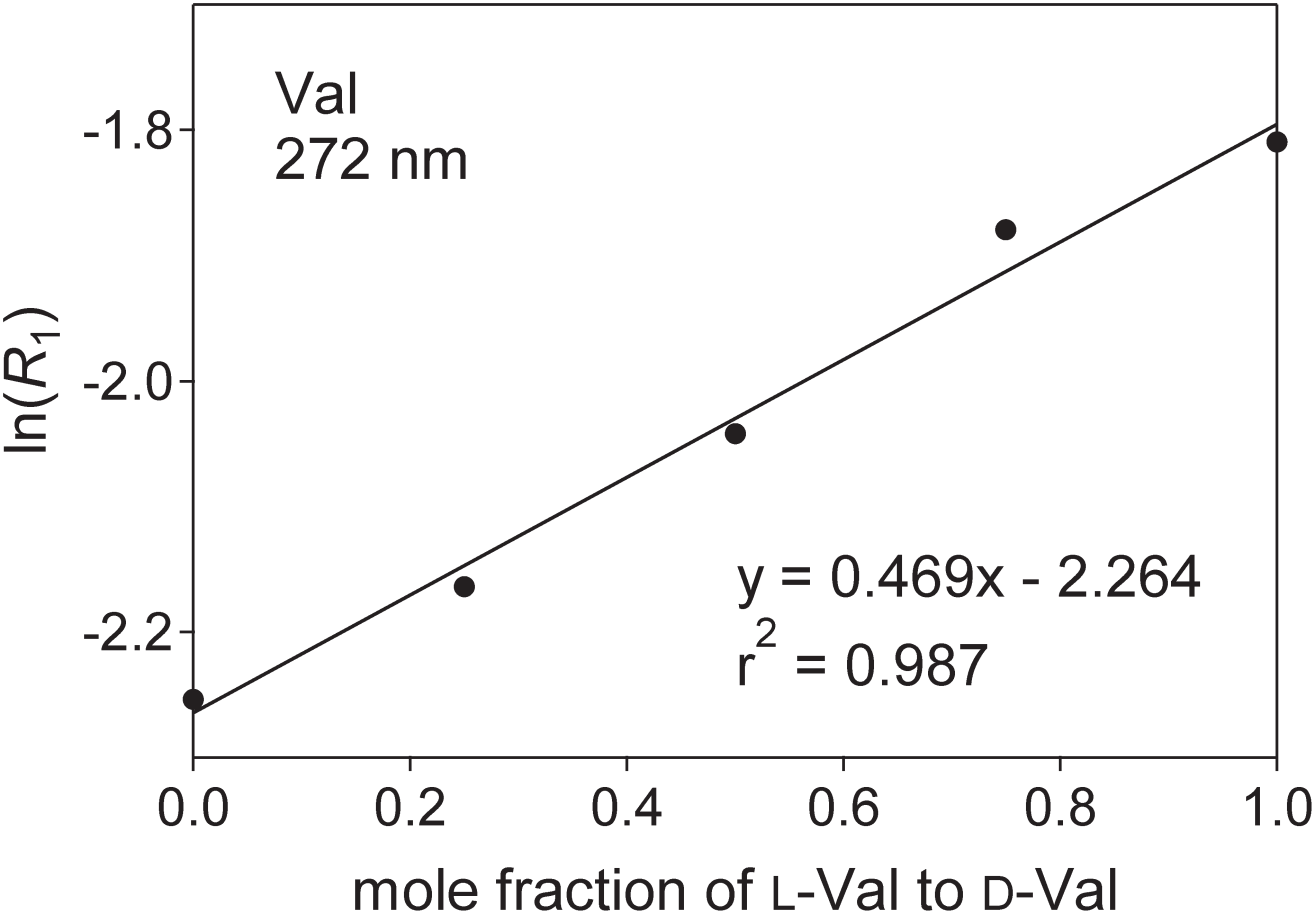
Fig. 5. Linear plot of ln(*R*_1_) for Na^+^(L-Trp)(Val) at 8 K *vs.* the mole fraction of L-Val to D-Val in solution. The relative abundance ratio *R*_1_ is derived by dividing the abundance of Na^+^(L-Trp) by the abundance of the precursor ion in the product ion spectrum measured by photoexcitation at 272 nm at 8 K for Na^+^(L-Trp)(Val).

To construct a calibration curve for quantifying the Ala enantiomer in solution, the product ion spectra at photoexcitation of the hydrogen-bonded clusters at 265 nm were measured for several L- and D-Ala enantiomeric mixtures, with L-Ala to D-Ala mole fractions of 1.00, 0.75, 0.50, 0.25, and 0.00. The 1.00 mol fraction of L-Ala to D-Ala represents an L-Ala solution containing L-Trp, with the product ion spectrum of Na^+^(L-Trp)(L-Ala) displayed in Fig. 1b. The 0.00 mol fraction of L-Ala to D-Ala represents a D-Ala solution containing L-Trp, with the product ion spectrum of Na^+^(L-Trp)(D-Ala) displayed in Fig. 1a. From the product ion spectra at photoexcitation of the mixtures at 265 nm, the ratio of the relative abundance *R*_1_ is derived by dividing the abundance of Na^+^(L-Trp) by the abundance of the precursor ion, and the natural logarithm of *R*_1_ is plotted as a function of the mole fraction of L-Ala to D-Ala in Fig. 4. A linear relationship is observed with an intercept of −2.069, a slope of −0.921, and a correlation coefficient (*r*^2^) of 0.989. For Val enantiomers that were examined using photoexcitation of 272 nm, the intercept, slope, and *r*^2^ of the linear relationship are −2.264, 0.469, and 0.987, respectively, as shown in Fig. 5.

Therefore, the mole fraction of L-enantiomer in solution was determined from *R*_1_ derived from the single product ion spectrum. The key dissociation parameters, the excitation energy and temperature of the gas-phase ions, were controlled using a single-nanosecond UV laser pulse and a cold ion trap, respectively.^33)^ However, the relative abundance of the product and precursor ions, *R*_1_, is dependent on the laser power and the spatial overlap between the ion packet and the laser pulse. Thus, the linear *R*_1_ relationship should be calibrated with both the L- and D-enantiomers for each experimental condition.

**Figure 6:**
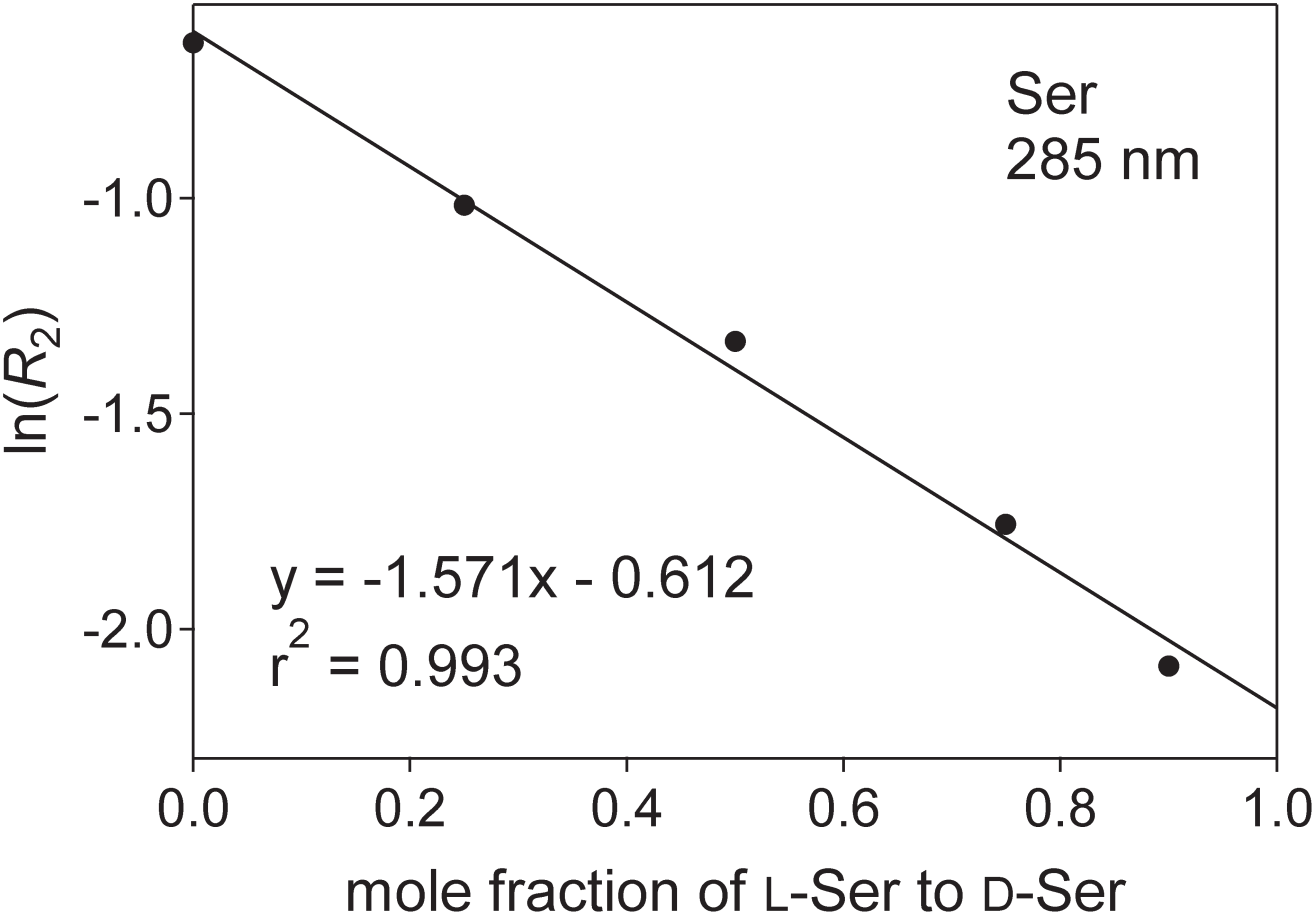
Fig. 6. Linear plot of ln(*R*_2_) for Na^+^(L-Trp)(Ser) at 8 K *vs.* the mole fraction of L-Ser to D-Ser in solution. The relative abundance ratio *R*_2_ is derived by dividing the abundance of the CO_2_-eliminated ion by the abundance of Na^+^(L-Trp) in the product ion spectrum measured by photoexcitation at 285 nm at 8 K for Na^+^(L-Trp)(Ser).

The relative abundance of product ions formed *via* one-photon absorption, *R*_2_, is independent of the laser power and the spatial overlap between the ion packet and the laser pulse. Ser enantiomers are identified using *R*_2_, as shown in Fig. 3c. To construct a calibration curve for quantifying the Ser enantiomer using 285 nm photoexcitation, the product ion spectra of L- and D-Ser mixtures were obtained, as for Ala and Val. The natural logarithm of *R*_2_ is plotted as a function of the L-Ser to D-Ser mole fraction in Fig. 6, where *R*_2_ is derived by dividing the abundance of the CO_2_-eliminated ion by the abundance of Na^+^(L-Trp) in the product ion spectrum. A linear relationship for photoexcitation at 285 nm was observed. Therefore, photoexcited Trp recognizes amino acid enantiomers through intermolecular hydrogen bonds.

## CONCLUSION

Enantiomer selectivity among amino acids was investigated using UV photoexcitation of cold gas-phase hydrogen-bonded clusters generated *via* electrospray ionization and collisional cooling. Amino acid enantiomers were identified based on optical properties and enantioselective reactions of hydrogen-bonded Trp within the clusters. Ala, Val, and Ser enantiomers in solution were quantified from their relative abundances in single product ion spectra that were obtained by the photoexcitation of hydrogen-bonded Trp within the clusters at 265, 272, and 285 nm, respectively. Enantioselective phenomena of amino acids were observed in studies regarding chemical evolution in interstellar molecular clouds. Insights into the molecular characteristics and their origins regarding chemical evolution would be valuable for analytical method development.

## Notes

Mass Spectrom (Tokyo) 2021; 10(1): A0097
